# Effect of Surface Modification on the Properties of Polypropylene Matrix Reinforced with Coir Fibre and Yam Peel Particulate

**DOI:** 10.1155/2021/8891563

**Published:** 2021-02-10

**Authors:** Adeolu A. Adediran, Oluwatosin A. Balogun, Abayomi A. Akinwande, Fredrick M. Mwema, Olanrewaju S. Adesina, Adeniyi Olayanju

**Affiliations:** ^1^Landmark University SDG 9 (Industry, Innovation and Infrastructure Research Group), Omu-Aran, Kwara 1001, Nigeria; ^2^Department of Mechanical Engineering, Landmark University, Omu-Aran, Kwara 1001, Nigeria; ^3^Department of Metallurgical and Materials Engineering, Federal University of Technology, Akure, Ondo 704, Nigeria; ^4^Department of Mechanical Engineering, Dedan Kimathi University of Technology, Nyeri 10143, Kenya

## Abstract

Polypropylene composites reinforced with coir fibre and yam peel particulate were produced using compression moulding machine. Treated and untreated coir fibres were used; 1.5 M NaOH was used for the treated coir fibres. Yam peel was grouped into two, treated and untreated; the treated was modified using 1 M solution of NaOH and HCl in the proportion of 30% and 70%, respectively. The yam peel which was sun-dried for 14 days was pulverized and sieved to −45 *µ*m. Samples were developed using treated and untreated reinforcements (TCF/YPP and UCF/YPP) at constant coir fibre proportion (15%) and varied amount of yam peel particulate (2, 4, 6, and 8 wt.%). The hybrid composite samples developed were probed for mechanical properties and thermal and wear behaviour. The level of particles agglomeration at the fibre-matrix interface was examined using scanning electron microscope. The results show that sample reinforced with treated 4 wt.% coir fibre and yam peel particulate had optimum mechanical properties. However, the thermal conductivity of composite samples increased with fibre addition. All composite samples developed had better resistance to abrasion when compared to the control sample.

## 1. Introduction

After energy generation distribution and conservation, one of the most important problems in the world is safeguarding of the environment. New plans are being made every day in a bid to produce environmentally friendly materials which are not harmful to both humans and the environment at large. Owing to this reason, researchers have diverted towards the utilization of natural fibres for the production of eco-friendly composites as a substitute over synthetic fibres being employed in the past. These fibres are adopted as additives in polymer resin for multiple advanced applications on account of their light weight, sustainability, renewability, high modulus, and non-toxic nature [1, 2]. Natural fibres are characterized with distinct properties. For instance, they are cheap, available in abundance, and renewable. Also, they possess the capacity to absorb carbon dioxide (CO_2_) from the atmosphere thereby improving the quality of oxygen available for human beings. Similarly, these fibres do not give off deleterious gas and the equipment employed in processing them is not abraded, of which the opposite is the case for synthetic fibres [3, 4].

The key disadvantage of natural fibres is their high flammability and their hydrophilic nature which curtail their efficiency as reinforcement in polymeric matrix. These fibres due to their inherent hydrophilic nature are characterized with high moisture absorption which leads to poor interfacial adhesion with the matrix. However, numerous researches have been carried out on modifying the surface of natural fibres in order to resolve these limitations which does not occur in the case of synthetic fibres [5–7]. Chemical treatments such as silane, benzylation, acrylation, mercerization, acetylation, isocyanates, permanganate, and alkali treatments have shown significant success in improving the interfacial adhesion between natural fibres and polymer matrix. This results in the formation of composite with ameliorated properties as observed in recent studies [8–11]. Recently, there has been a trend of utilizing two reinforcements in a polymer matrix usually referred to as hybrid composites. These new materials are developed in order to derive a certain desired property while also improving the overall properties of the hybrid composite. The idea was aimed at inducing a certain property as well as service performance by the use of another additive to overcome the deficiency of single filler [12]. Some researchers have focused on studying the influence of natural fibre along with additional filler which can be either synthetic or natural. Sakthivel et al. [13] employed luffa and coir fibres in the development of polypropylene based composite. The study highlights that the addition of these additives led to improvement in hardness, flexural rigidity, tensile strength, and impact characteristics while also exhibiting water absorption of 1%, indicating the effect of chemical treatment in achieving improved bonding at the fibre-matrix interface. The influence of chemical treatment on the characteristics of epoxy composite reinforced with palm kernel fibre and cassava peel particulate was reported by Oladele et al. [14]. The outcome of their study revealed 6 wt. % additive as the composition with the optimum property while identifying improvement in flexural strength, Young's modulus, and resistance to wear. Nevertheless, pure epoxy had better tensile strength than all hybrid composites developed. Also, Mark et al. [15] embedded carbonized coconut shell particles of various particle sizes as additive in polypropylene composite; the additive was added at 0–40% by weight of polypropylene matrix. The composite samples were produced by injection moulding process and the result showed increased hardness and tensile and flexural strengths. However, modulus of resilience and elongation at break declined with increasing carbonized coconut shell particulate content. Kwon et al. [16] proved that the use of kenaf fibre and corn rice husk in developing novel hybrid bio-composite had positive influence on the mechanical properties of polylactic acid. Further study on kenaf and aramid fibres as hybrid reinforcement in epoxy composites showed a low density and high void content in samples with high weight content of kenaf. Similarly, thickness swelling and water absorption were higher as the content of kenaf increased. This insinuates that reduced impact strength was obtained as kenaf content increased, due to the fact that impact strength of composites is affected by its water absorption characteristics [17]. The effect of coir fibre as an efficient reinforcing material in polymer matrix has been established in previous researches [18–20]. On the other hand, yam is an annual of perennial starchy staples which are in tuber form. It is mainly grown in West Africa, of which Nigeria carries a larger share of about 67% of the total yam produced throughout the world. A total of 44.1 million tonnes of yam is produced yearly in Nigeria [21]. After the edible part is consumed, the peel which is usually regarded as wastages is disposed and will eventually lead to more environmental disturbance. Literature on the use of yam peel as a reinforcing material in polymeric matrix is scarce. The use of yam peel as reinforcement would serve as a value addition to the waste material, which when left alone constitutes environmental burden in Nigeria. Also, the properties of the composites developed can be enhanced with this additive. This study considered it worthwhile to examine the combination of coir fibre and yam peel particulate as potent reinforcements in polypropylene which is a common thermoplastic matrix material. Also, the mix proportion of coir fibre and yam peel particulate that would yield the optimum mechanical, thermal, and wear properties would be determined. Furthermore, the result obtained would broaden the horizon of knowledge on the use of these additives in both treated and untreated state.

## 2. Materials and Methods

### 2.1. Materials

**Figure 1:**
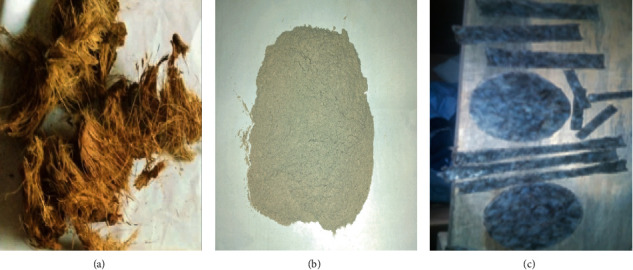
Images of (a) coir fibre, (b) YPP, and (c) test samples.

Materials used in this study include coir fibre which was gotten as a waste, from a local coconut store in Ibadan, Oyo State, Nigeria. Waste yam peel was collected from a local food canteen also in Ibadan, where yam peel was packed for disposal, while polypropylene, sodium hydroxide (NaOH), hydrochloric acid (HCl), and distilled water were procured from a local chemical store. The images of the coir fibre, yam peel particulate, and some of the test samples are shown in Figure 1.

### 2.2. Methods

#### 2.2.1. Processing of Coir Fibre

The coir fibres obtained from coconut store were washed with clean water and sun-dried for 5 days during the rainy season. Similar to the procedure employed in Mohammed et al. [22], the fibres were divided into two groups. Group one was left untreated while group two was treated in 1.5 M NaOH solution for 12 hours at room temperature (27°C), after which they were washed with distilled water to make sure of the total removal of NaOH remnants. Sun-drying of the fibre was effected for 7 days and nicked to a length of 30 mm and employed in the preparation of the samples [23, 24].

#### 2.2.2. Processing of the Yam Peel

Yam peel obtained from local food canteen was washed with clean water and divided into two groups; group one was left untreated while group two was treated using 1 M solution which contains the mixture of NaOH and HCl in the proportion of 30% and 70%, respectively. Yam peel was immersed in a beaker containing the mixed solution and placed in a shaker water bath operated at 50°C for 3 hours [12]. Sequel to this is the washing using distilled water to make sure of the total removal of both NaOH and HCl residues. The dried peel was sun-dried for 14 days, pulverized, and then sieved to a particle size of −45 *µ*m using laboratory sieve shaker [25].

#### 2.2.3. Composite Developments

The control and composite samples were produced utilizing compression moulding machine. Coir fibre (CF) and yam peel particulate (YPP) were mixed with polypropylene matrix in a fixed proportion as revealed in Table 1. Cast iron moulds employed were cleaned and rubbed with petroleum jelly to ensure easy removal of the samples from the mould. Production was carried out at a temperature of 165°C for 10 minutes. Compounding was effected for even distribution of additive in the matrix. Two sets of samples were produced; the first set was produced using untreated coir fibre and yam peel particulate (UCF/YPP) while the other set was produced using treated coir fibre and yam peel particulate (TCF/YPP).

### 2.3. Property Evaluation

#### 2.3.1. Melt Flow Index

The appraisal of melt flow index for each weight fraction was done in concert with ASTM D1238-13 [26]. The test was carried out using the melt flow indexer (MF12-203). Samples were placed within the die and a load of 2.16 kg was applied though a piston at a temperature of 230°C. The amount of material deposited in ten minutes was collected and used in evaluating melt flow index.

#### 2.3.2. Tensile Properties

Tensile properties of the control and the CF/YPP hybrid composite samples were determined in concert with ASTM D3039/D3039M-17 [27] by subjecting three samples of each weight fraction with dumbbell shape to test. A load cell of 10 kg was adopted to simulate a state of tensile loading on the samples. This was done to evaluate the tensile strength and modulus at break on each sample of 3 mm thickness and 150 mm gauge length. Evaluation of tensile properties was done using Instron Universal Testing Machine (3369 Series).

#### 2.3.3. Flexural Properties

Flexural properties for each weight fraction were appraised in congruence with ASTM D790-17 [28] standard utilizing Instron Universal Testing Machine (3369 Series). This test was carried out with a 0.3 mm/mm crosshead speed and a strain rate fixed at 10^−3^/s. Samples with dimensions of 150 × 50 × 3 (mm^3^) were subjected to test with the aim of assessing their flexural strength and modulus at break for each sample. Three matching samples were evaluated to determine their average which was adopted as the representative value for each weight fraction.

#### 2.3.4. Impact Strength

Samples resistance to impact load was assessed in concert with ASTM D256–10 [29] via Charpy impact testing machine. Samples with dimensions of 80 × 10 × 3 (mm^3^) were notched at the midpoint and appropriately positioned while ensuring a distance of 60 mm was maintained between the supporting lines. Samples were fractured with the aid of suspended pendulum swing. Initial values on the gauge were recorded prior to fracturing of samples and the final reading after fracture was also taken. Three matching samples were evaluated to determine the mean values for each weight fraction.

#### 2.3.5. Wear

Taber abraser was utilized in abrading the surface of samples. Wear test was executed in conformity with ASTM F732-17 [30]. This was accomplished by mounting samples on the rotating platform usually fixed at a speed of 1000 rpm for a period of 20 min. Samples surfaces were exposed to a rub-wear action and this generated a circular pattern as the abrasion proceeded. Initial and final weight were measured using the analytical weighing balance and wear loss was computed employing expression (1). Three identical samples were appraised to determine their average.

(1) $$\begin{array}{c} W=W_{1}-W_{2}, \end{array}$$

where *W* is wear loss; *W*_1_ is the weight of sample before abrasion; and *W*_2_ is the weight of sample after abrasion.

#### 2.3.6. Thermal Conductivity

Modified Lee's disk apparatus was employed in appraising the thermal conductivity of the developed composite. This test was conducted in commensuration with ASTM E1530–19 [31]. Samples were evaluated at elevated temperature of 50–80°C which is lower than the degrading temperature of the materials employed. Equation (2) was employed in computing the thermal conductivity for each weight fraction.

(2) $$\begin{array}{c} T=\;\frac{\text{mcp}\left(\emptyset_{1}-\emptyset_{2}\right)4\;d}{\pi \;dD^{2}\left(T_{1}-T_{2}\right)t}, \end{array}$$

where *T* is thermal conductivity; *w* is mass of the metallic disc; cp is specific heat capacity of disc; ∅_1_ is initial temperature of disk 2; ∅_2_ is final temperature of disk 2; *D* is the diameter of each sample; *d* is thickness of each sample; *T*_1_ is initial temperature of disk 1; *T*_2_ is final temperature of disk 1; and *t* is the time taken for heat to attain steady state.

## 3. Results and Discussion

The properties of the coir fibre were examined to determine its inherent characteristics such as moisture content (9.51%), density (1.25 g/cm^3^), diameter (250 *µ*m), tensile strength (155 MPa), Young's modulus (5.4 GPa), and elongation (23.75%).  Table 2 shows the chemical composition of coir fibre and it was revealed that the chemical treatment caused a depreciation in the amount of hemicellulose and lignin. These constituents are responsible for the fibre degradation, hydrophilic characteristic, and poor interfacial adhesion with the intending polymeric matrix [32]. Improved interaction between matrix and reinforcement can be enhanced via chemical treatment to selectively depreciate the amount of the hemicellulose and lignin while improving the rough surface of the fibres. This treatment modifies the hydrophilic nature of the fibre to ensure good adhesion with the matrix which is highly hydrophilic [33]. Yam peel as shown in Table 2 contains compounds such as SiO_2_ and also Fe_2_O_3_ usually regarded as good reinforcers with potential of improving mechanical properties of the matrix material.

### 3.1. Melt Flow Index

**Figure 2:**
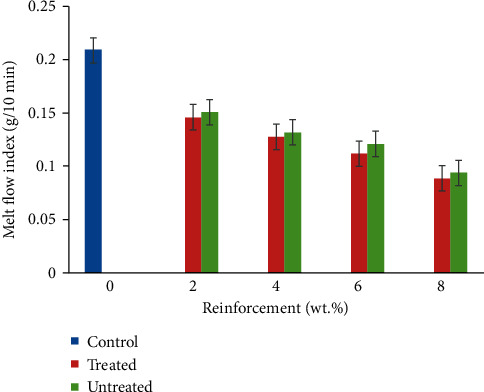
Variation in melt flow index of coir fibre and yam peel particulate polyethylene composite.

The result obtained for melt flow index of CF/YPP composite is shown in Figure 2. Embedding of CF/YPP was noted to lower melt flow index of the resulting composite which infers an increased viscosity [34]. Linear reduction was observed from 2 to −8 wt.% CF/YPP; this can be linked to the combined effect of coir fibre and particulate addition. In [35], a similar result was recorded after they incorporated coir fibre into thermoplastic cassava starch/polylactic acid. Reduced melt flow index was discovered with increasing amount of fibres when samples were examined at a temperature of 190°C and applied load of 2.16 kg. The result observed may ensue on the account of stiffening effect created by the presence of fibre and particulate which impeded the rate at which the melted polymer flowed. The study of [36, 37] also confirmed this proposition. However, [38] showed that the addition of hydrolysed powder coating increased melt flow index of polypropylene to 40 wt.% reinforcement addition. As the YPP content increases, there is more propensity of particle agglomeration and collision as stated by [39, 40]. Composite samples produced treated fibres and treated samples had deteriorated flow characteristics compared to their counterparts this is attributable to the surface roughness of fibres which emanated from the reduction of waxy cuticles and impurities present on fibre surfaces [41].

### 3.2. Tensile Strength at Break

**Figure 3:**
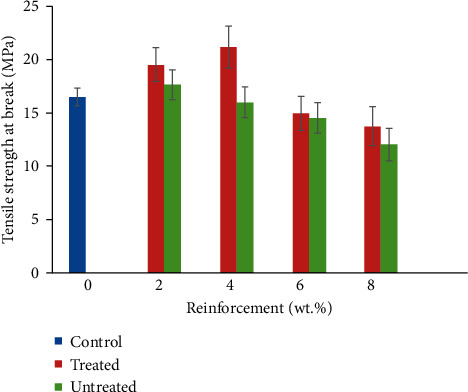
Variation in tensile strength of coir fibre and yam peel particulate polyethylene composite.

This property was examined to know the maximum load that the composite samples can withstand under tensile load before breaking. Variation of CF/YPP composite to tensile strength is shown in Figure 3. Sample reinforced with 4 wt.% TCF/YPP emanated with the optimum values showing a 32.73% enhancement compared to pure polypropylene. Tensile strength of a material depicts its resistance to tensional stress when the load is applied in a particular direction. In the current study, it was observed that tensile strength increased from 2 to 4 wt.% TCF/YPP followed by a reduction from 6 to 8 wt.% TCF/YPP while for the untreated samples, this property reduced in a downward trend from 2 to 8 wt.% UCF/YPP. Tensile strength increased based on the efficiency of CF/YPP to effectively reinforce polypropylene matrix up to 4 wt.% reinforcement addition, which leads to effective stress transfer within the matrix. Further increase in reinforcement content induced inability of the fibres and yam peel particulate to effectively aid stress transfer [42]. The common trend observed was that TCF/YPP reinforced composites had higher tensile strength at break than UCF/YPP reinforced composites. This evoked the fact that surface modification of natural fibres improves their performance when incorporated into matrix material. The result obtained is in tandem with previous research work of [16, 43]. Natural fibres acquired from plants are hydrophilic owing to their lignocellulose nature which yields highly polarized hydroxyl group. This translates into high water absorption characteristics, degrading of the fibres which initiate poor interfacial adhesion at the fibre-polymer interface, and hence loss of mechanical property [44]. From their study, the authors of [22] concluded that swelling of fibres as a result of moisture absorption leads to degradation of natural fibres. Therefore, the removal of lignin content is essential since lignin is an unreactive constituent of natural fibre that keeps water content. In addition, treatment of these agricultural wastes is deemed important to improve interfacial adhesion and mechanical property [45, 46].

### 3.3. Tensile Modulus at Break

**Figure 4:**
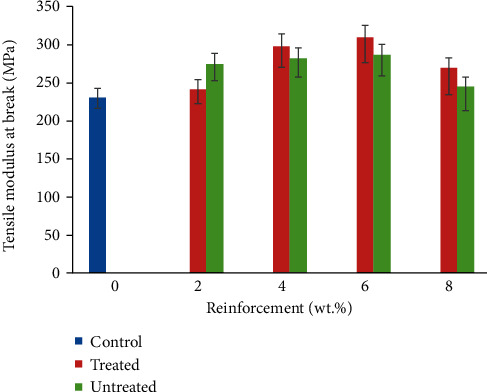
Variation in tensile modulus of coir fibre and yam peel particulate polyethylene composite.

Figure 4 highlights the response of polypropylene based composite as a function of CF/YPP addition. Increase in the filler content was observed to prompt increased tensile modulus. This property increased from 231 MPa at 0 wt.% CF/YPP to 310 MPa at 6 wt.% CF/YPP which begat a 34.20% increase. This behaviour may ensue on account of rigid phase inclusion which invoked stiffness in the resulting composite. The research study of [47] shows an upward trend in tensile modulus from 526 MPa in control sample to 820 MPa at 40 wt.% eggshell addition. Also, the authors of [48] also confirmed the increasing effect of particulate reinforcement on the modulus of polypropylene matrix with the incorporation of particulate filler of 8.4 µm. In this study, the common trend observed was an increase in tensile modulus of treated ones except in the case of 2 wt.% CF/YPP where untreated reinforcements had higher value. This depicts that at lower particulate addition untreated filler had better reinforcing characteristics. 6 wt.% CF/YPP had the optimum performance. This is in agreement with the study of [49]. Also, the findings of [50] showed that increase in particulate content in the presence of pineapple leaf fibre improved tensile modulus which buttressed the result obtained in the present study. The treatment enhances the modulus of both the fibre and reinforcement showing the effect of chemical treatment which modified fibre surface and could possibly favour higher modulus observed. A previous study has shown that chemical treatment is a potent way of reducing lignin content in a natural fibre which in turn increases the roughness of cellulose and at the same time the surface area of cellulose ready for contact with the matrix [51]. In Table 3, YPP is made up of compounds such as Fe_2_O_3_ and SiO_2_ which are present in considerable amount. These elements can also contribute to the enhancement observed. The utilization of reinforcement in polymeric material can serve as a strengthening mechanism, in that the additives are impediments to the free movement of dislocation. Therefore, when a force is applied on the material, the reinforcements hinder the movement of these dislocations which results in higher strain and stiffness [14]. In addition, the absence of these impediments in the control sample led to a lower value of modulus observed. In recent years, agricultural wastes have been making headway due to their availability, non-toxic nature, and also low-cost implication. The use of these materials in polymeric matrix is a practical, economically, and constructive viable waste management process.

### 3.4. Flexural Strength at Break

**Figure 5:**
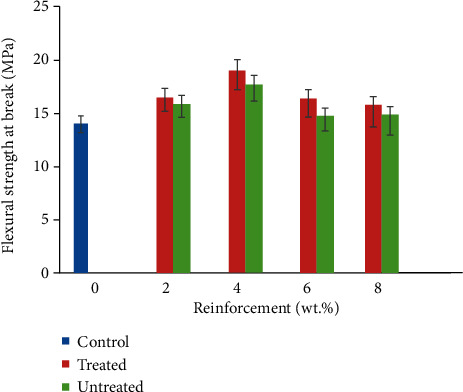
Variation in flexural strength of coir fibre and yam peel particulate polyethylene composite.

**Figure 6:**
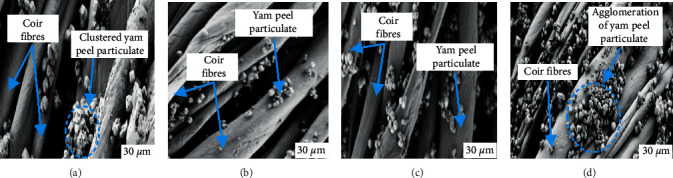
Morphology of (a) 2 wt.% CF/YPP, (b) 4 wt.% CF/YPP, (c) 6 wt.% CF/YPP, and (d) 8 wt.% CF/YPP samples at the reinforcement-matrix interface.

Flexural test estimates the amount of load under a three-point bending state. The result obtained is shown in Figure 5. It is seen that 4 wt. % TCF/YPP had the maximum flexural strength which is 35.68% increase over the strength of the control sample. At 6 wt.% CF/YPP addition reduction in flexural strength was noted; this behaviour is traceable to increased agglomeration of particles at that weight percent as indicated by the SEM micrograph as observed in Figure 6. Increase in YPP from 6 to 8 wt.% facilitates more agglomeration which in turn culminates strength reduction at that weight percent. Also, increased reinforcements at 6–8 wt.% has lessened the matrix to a high extent, which reduces the reinforcing capabilities of coir fibre and yam peel particulates; this results in improving ease of slip-off and eventual fracture at the application of stress [44]. The common trend observed between tensile and flexural strength at break was that optimum values were obtained at 4wt.%, after which a decline in these properties was recorded. The authors of [49] stated that the addition of particulate chitosan to high-density polyethylene matrix at 4% by weight of matrix result in coalescence of the particulate reinforcement and further increase led to congestion in the amount of filler and thus reduced flexural strength was discovered. This justified the result gotten in this study. Samples developed with modified CF/YPP had better performance than those used without modification. For instance, at 4 wt.% CF/YPP flexural strength revealed values of 19.05 MPa and 17.68 MPa for treated and untreated samples, respectively. This seems to suggest that surface modification improved resistance of every sample to bending load due to improved adhesion [52]. The high rate of water absorption has been lowered along with the reduction in propensity of fibre degradability while in service [53]. Furthermore, increased service life and mechanical properties ensued owing to reduction in the rate at which inherent cracks propagate when bending stress is applied on the developed composite.

### 3.5. Flexural Modulus

**Figure 7:**
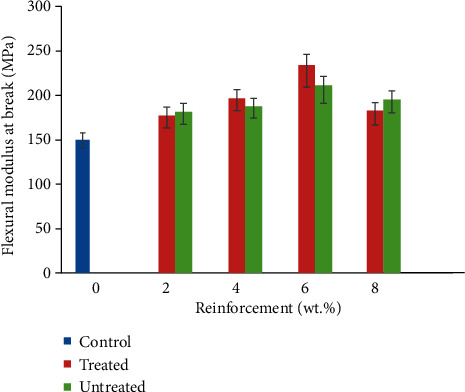
Variation in flexural modulus of coir fibre and yam peel particulate polyethylene composite.

From Figure 7, it is noted that flexural modulus of CF/YPP composite increases with CF/YPP addition from 2 to 6 wt. %; after this is a reduction at 8 wt.%. The common trend observed for tensile and flexural modulus optimum values was noted at 6 wt.% which implies that samples reinforced with 6 wt.% have more resistance to deformation and deflection than other samples developed. This corroborates with the study of [54] that observed an increased flexural modulus of epoxy composite reinforced with sisal fibre/calcined and uncalcined egg shell particulate. The result indicates that this property increases with increasing content of additive. In this study, 6 wt. % gave the best performance of 234 MPa, highlighting a 56% increase compared to the reference sample. Increased modulus up to that weight percent can occur by dint of CF/YPP addition; particulate reinforcement coupled with fibre addition can improve to a large extent the stiffness of the resulting composite [55]. Also, higher flexural modulus was observed for treated reinforcements over the untreated ones due to higher aspect ratio in treated reinforcements which influences the stiffness of the resulting composites positively, as modulus of the composite is dependent on the modulus of the reinforcing phase [56]. However, at higher weight fraction of reinforcement there is tendency of these particles to agglomerate rather than effective filling pores; they cause more porosity in the developed composite. Similarly, the authors in [57] studied the influence of cow hair on flexural modulus of polyester; the result shows that the incorporation of this fibre improves flexural modulus up to 20 wt. %. Chemical treatment given to the fibre does not necessarily result in higher modulus than the untreated one. Untreated 20 wt.% cow hair gave the best flexural modulus. Conversely, treated 6 wt. % YPP gave the best performance, though at high weight fraction of CF (25 wt.%). This study further avers the claim that recycling of agro waste into polymer matrix is a good method of improving the mechanical properties of the intending polymers in both treated and untreated state. This result justifies the utilization of CF/YPP as effective reinforcement in polypropylene matrix.

### 3.6. Impact Strength

**Figure 8:**
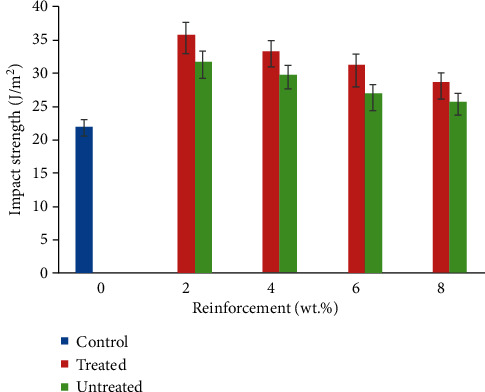
Variation in impact strength of coir fibre and yam peel particulate polyethylene composite.

The result of impact strength of CF/YPP composite is shown in Figure 8. The optimum value was discovered at 2 wt.% CF/YPP. All composite samples were observed with better impact resistance than the control, as a consequence of CF addition which serves as an obstacle to crack propagation and also affects medium of load transfer in the polypropylene matrix Pure polypropylene matrix has shown good resistance to impact load as reported by previous researches [58]. However, increase in YPP content led to a reduction in the polypropylene matrix which culminated in depreciation in the toughness of the composite. According to [59], composite with the highest impact strength usually possesses the lowest stiffness and vice versa. This claim was justified in this study. The 2 wt. % had the lowest tensile and flexural modulus of all the composite samples; however, it emanated with the highest impact resistance. The presence of YPP led to increase in the rigidity of the composite as its weight content increased from 2 to 8 wt. %. At 8 wt.%, due to the high stiffness of the composite, a huge amount of stress was transferred from the matrix to the reinforcements thereby resulting into low impact property. The 2 wt. % TCF/YPP was observed with 62.95% improvement over the control sample. Treatment of the CF and YPP resulted in higher impact strength than those that were not treated showing 12.88%, 12.33%, 16.40%, and 11.91% over their untreated counterparts at 2 wt. %, 4 wt. %, 6 wt.%, and 8 wt.% CF/YPP, respectively. This may be attributed to the reduction in the hydrophilic behaviour occasioned by NaOH and HCL treatments which enhance the adhesion at the matrix-reinforcement interface. Therefore, improved compatibility and toughness are achieved in the resulting composite. It is noted in [60] whose research work showcases the influence of chemical treatment on the impact properties of polypropylene composite. It was discovered that samples reinforced with treated coir and palm fibres had better impact resistance than those that were not treated. The result of this study substantiates the findings of [59] that concluded that increase in particulate content ensued reduced impact strength. The study of [48, 49] also serves as a confirmation to this assertion. In addition, the SEM images from Figures 6(a)–6(d) are also characterized with increase in agglomeration of YPP at the matrix-fibre interface which could also be responsible for the reduction in impact strength.

### 3.7. Wear

**Figure 9:**
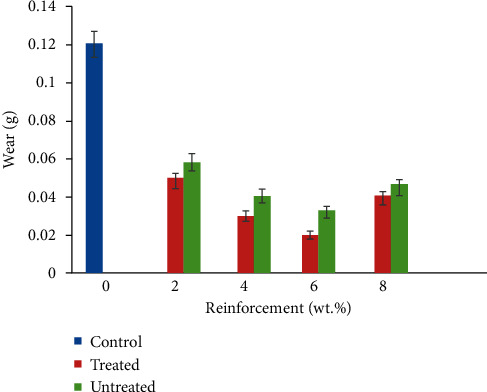
Variation in wear of coir fibre and yam peel particulate polyethylene composite.

The behaviour of CF/YPP reinforced polypropylene composite in terms of wear loss is as represented in Figure 9. The result obtained highlights that these additives led to reduction in wear loss and increase in the resistance of the composite samples to abrasion. This outcome is likely to ensue due to coefficient of friction occasioned by reduced frictional force. Previous studies have emphasized the role of natural fibres in generating low coefficient of friction and reducing the contact area of the polymeric material exposed to the abrading medium [61]. However, the authors in [14] pointed out the effectiveness of both treated and untreated palm kernel fibre and particulate cassava peel in improving the wear properties of epoxy composite, noting that treated composite sample at higher weight fraction of particulate addition had the optimum performance. Similarly, the study on polypropylene reinforced with treated *Dombeya buettneri* and graphite also confirmed that the addition of second phase improved wear characteristics [12]. This study further buttresses the fact that both treated and untreated CF/YPP had a significant impact in improving the resistance of polypropylene to abrasion. The 6 wt.% TCF/YPP had the optimum performance showing a 476.2 % depreciation in wear loss compared to pure polypropylene. Increase in wear property may be linked to increase in modulus which is a factor of modulus fibre volume and hardness [14, 57]. The graph obtained for wear follows the same trend as that of modulus; this ratifies the relationship stated by Lai et al. [55]. 2 wt. % UCF/YPP had the highest wear loss among the composite produced, with a wear value below that of control. This infers that in an application improved wear is of high importance. The composite developed can produce better service performance than the pure polypropylene.

### 3.8. Thermal Conductivity

**Figure 10:**
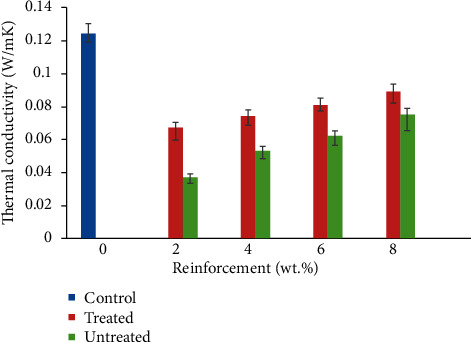
Variation in thermal conductivity of coir fibre and yam peel particulate polyethylene composites.

Thermal conductivity of a material measures the rate of heat flow across the material through a unit thickness when it is subjected to change in temperature [62]. High thermal conductivity in a material implies low thermal insulation characteristics. A previous study has linked the thermal conductivity of a composite to the inherent properties of starting materials and the amount of pores generated in the composite [63]. Since air is a good thermal insulator, increase in the amount of air spaces or voids would give rise to reduced thermal conductivity. Lower volume of pores in a material infers the close packing of the constituent and hence increased thermal conductivity [64]. Many natural fibres have displayed high aspect ratio, cellulose content, and a very tiny size of lumen which makes them exhibit good thermal insulation characteristics as well as being light weight and offers versatility in various engineering applications [64]. In this study, Figure 10 shows that 8 wt. % TCF/YPP had the highest thermal conductivity in the developed composite samples. The presence of treated natural fibres in polymers tends to increase thermal conductivity as compared to that of the untreated due to the fact that the thermal conductivity of individual fibre and particulate has been increased as a result of lignin and wax removal. The cumulative effect of treated yam peel at 8 wt.% addition may give rise to the most significant thermal conductivity improvement observed at that weight percent as compared to others samples [65]. This property significantly increases in YPP for both treated and untreated CY/YPP composites it substantiates the research work of [66] that noted reduced thermal conductivity of polypropylene with increasing particulate rice straw. Treatment of fibres reduces the diameter of fibres which caused increased aspect ratio. This occurrence may be linked to removal of waxy cuticles lignin and hemicellulose which reduces the rigidity of the interfibrillar regions thereby causing easy re-arrangement of the fibrils [67]. The crystallinity index of the treated fibres is improved owing to dissolution of the cementing material in NaOH medium resulting into enhanced packing of the cellulose network and hence increased thermal conductivity [68]. All untreated samples had lower thermal conductivity than the treated ones, agreeing with the study of [69]. In another report, the authors in [70] concluded that phenol formaldehyde reinforced with treatment of oil palm fibres had higher thermal conductivity than those produced with untreated fibres. The 4 wt.% UCF/YPP composite being the sample with the lowest thermal conductivity shows a 235.13% reduction compared to pure polypropylene, insinuating the fact that this sample had the optimum thermal insulation property.

### 3.9. Scanning Electron Microscopy

The scanning electron micrograph of the hybrid composite in Figures 6(a)–6(d) reveals the level of agglomeration of particles at fibre surface in composite samples reinforced with 2 wt. %, 4 wt. % 6 wt. %, and 8 wt. %, respectively. At 2–4 wt. %, low level of agglomeration was observed at the fibre interface which complements the result obtained for the mechanical properties. Higher volume of YPP results in increased agglomeration and reduction in mechanical property [71]. This insinuates that the use of YPP as reinforcement in a matrix material should not exceed 4 wt.%.

## 4. Conclusions

Coir fibre and yam peel particulate hybrid composite was produced using compression moulding machine, and the samples were probed for tensile, flexural, and impact strength; other properties include hardness, wear, and thermal conductivity [71]. The result obtained showed the following:

- Chemical treatments given to the reinforcements were effective in reducing the lignin and hemicellulose content of fibres, which gave rise to improved properties as compared to the untreated ones when incorporated into polymer matrix.
- Treated 4 wt.% CF/YPP is effective in enhancing the tensile and flexural strengths. Proportion of reinforcements greater than this is detrimental to tensile and flexural strengths. Tensile and flexural modulus were improved up to 6 wt.% CF/YPP, after which depreciation in these properties was observed.
- Impact strength of the produced composite showed a peak value at 2 wt.% TCF/YPP; however, consistent reduction in impact property was noted with increasing amount of YPP.
- The 4 wt.% TCF/YPP reinforced polypropylene composite sample had the optimum mechanical properties. Higher weight fractions of reinforcements led to agglomeration of YPP in the matrix-fibre interface and loss of mechanical properties.
- Incorporation of CF/YPP into polypropylene matrix led to reduction in wear resistance in all samples produced, with the optimum value observed at 6 wt.% TCF/YPP.
- Blending of UCF/YPP as reinforcements in polypropylene gave rise to improved thermal insulation which makes the composite samples a better candidate for household applications such as wall sockets, insulating pipes, and ceiling materials.

## Figures and Tables

**Table 1:** Proportion of materials employed in composite production.

Sample designation	CF (%)	YPP (%)	Polypropylene (%)
0	0	0	100
2	15	2	83
4	15	4	81
6	15	6	79
8	15	8	77

**Table 2:** Compositional analysis of coir fibre.

Constituent	Treated	Untreated
Cellulose (%)	51.85	42.54
Hemicellulose (%)	37.43	44.47
Lignin (%)	0.11	0.22
Others (%)	10.61	12.77

**Table 3:** XRF analysis of yam peel particulate.

Compounds	(wt.%)
SO_3_	0.32
SiO_2_	0.52
TiO_2_	0.48
Cl	3.14
Fe_2_O_3_	2.57
Br	0.23
CuO	0.51
Dy_2_O_3_	1.78
RuO_2_	5.04
Organic matter	85.41

## Data Availability

The data used to support the findings of this study are included within the article.
